# Physical Activity and Modernization among Bolivian Amerindians

**DOI:** 10.1371/journal.pone.0055679

**Published:** 2013-01-31

**Authors:** Michael Gurven, Adrian V. Jaeggi, Hillard Kaplan, Daniel Cummings

**Affiliations:** 1 Integrative Anthropological Sciences Unit, Department of Anthropology, University of California Santa Barbara, Santa Barbara, California, United States of America; 2 Sage Center for the Study of the Mind, University of California Santa Barbara, Santa Barbara, California, United States of America; 3 Department of Anthropology, University of New Mexico, Albuquerque, New Mexico, United States of America; Indiana University, United States of America

## Abstract

**Background:**

Physical inactivity is a growing public health problem, and the fourth leading risk factor for global mortality. Conversely, indigenous populations living traditional lifestyles reportedly engage in vigorous daily activity that is protective against non-communicable diseases. Here we analyze physical activity patterns among the Tsimane, forager-horticulturalists of Amazonian Bolivia with minimal heart disease and diabetes. We assess age patterns of adult activity among men and women, test whether modernization affects activity levels, and examine whether nascent obesity is associated with reduced activity.

**Methods and Findings:**

A factorial method based on a large sample of behavioral observations was employed to estimate effects of age, sex, body mass index, and modernization variables on physical activity ratio (PAR), the ratio of total energy expenditure to basal metabolic rate. Accelerometry combined with heart rate monitoring was compared to the factorial method and used for nighttime sampling. Tsimane men and women display 24 hr physical activity level (PAL) of 2.02–2.15 and 1.73–1.85, respectively. Little time was spent “sedentary”, whereas most activity was light to moderate, rather than vigorous. Activity peaks by the late twenties in men, and declines thereafter, but remains constant among women after the early teens. Neither BMI, fat free mass or body fat percentage are associated with PAR. There was no negative effect of modernization on physical activity.

**Conclusions:**

Tsimane display relatively high PALs typical of other subsistence populations, but of moderate intensity, and not outside the range of developed populations. Despite rapidly increasing socioeconomic change, there is little evidence that total activity has yet been affected. Overweight and obesity are more prevalent among women than men, and Spanish fluency is associated with greater obesity in women. The lack of cardiovascular disease among Tsimane is unlikely caused by activity alone; further study of diet, food intake and infectious disease is needed.

## Introduction

An active lifestyle is touted as one of the most important requirements for physical fitness and adult cardiovascular health [1]. Being sedentary is an independent risk factor for obesity, Type 2 diabetes, heart disease, dementia and other health conditions [2], [3], [4]. Physical inactivity has even been identified as a “pandemic” and the fourth leading risk factor for global mortality [1]. Despite the importance of physical activity for reducing morbidity and mortality and for promoting healthy aging, two-thirds of U.S. adults age 18+ never engage in vigorous leisure-time physical activities lasting 10+ minutes per week, and only 25% engage in such activity 3+ times per week [5]. Only 18% of U.S. adults engage in aerobic activity of at least moderate intensity for 150+ minutes per week and muscle-strengthening activities at least twice per week, as recommended as a key *Healthy People 2020* objective [6].

Unlike urban adults in the developed world, individuals living in subsistence societies typical of our preindustrial past are believed to have very active lifestyles [7], [8]. Preindustrial societies are also noteworthy because of the presumed scarcity of non-communicable diseases such as heart disease and diabetes [9]. Tsimane forager-horticulturalists of the Bolivian Amazon embody this pattern, showing few signs of obesity, diabetes, hypertension, atherosclerosis and coronary heart disease [10]. One hypothesis for this unique epidemiological profile is that a physically demanding subsistence lifestyle and low calorie diet may be critical for maintaining healthy metabolism, favorable body mass, blood lipids and cardiorespiratory health. To date, there has been no study of physical activity over the life course among a subsistence population with minimal labor-saving technology and high pathogen load like the Tsimane. In other subsistence groups where activity levels and energy expenditure have been estimated, sample sizes are often small and of restricted age ranges, e.g. [11], [12]. Epidemiological profiles in other subsistence populations are also lacking, making it difficult to establish empirical links between morbidity, functional status, and aging. This paper thus fills an important gap by documenting daily physical activity profiles across the life course and by examining effects of sex, seasonality, regional variation and modernization among a large sample of Tsimane adults age 20+. In doing so, we address four important research questions.

First, we ask whether Tsimane lifestyle is best characterized by light to moderate or moderate to vigorous activity. It is usually assumed that adults in subsistence societies are vigorously active, and that activity is a primary reason for why cardiovascular and other chronic non-communicable diseases are minimal [9]. However, intense subsistence activities may be of brief duration, or interspersed with days of rest, especially in less nomadic societies [11]. Disability from illness and injury may also depress physical activity in traditional societies [13]. Determination of activity levels in a population largely free of obesity and coronary heart disease would be instructive, especially in light of recent CDC and WHO recommendations promoting a mix of moderate and vigorous aerobic and muscle-strengthening activities. Contrary to assumptions, activity might be primarily of moderate intensity, rather than in line with these recommendations. If physical activity profiles overlap considerably with those in industrialized societies where CVD and diabetes are prevalent, factors other than activity may be better candidates for limiting chronic disease. For example, reduced caloric intake, diets high in potassium and omega-3 fatty acids, low psychosocial stress due to kin-based residence and frequent transfers across households, and an immune system shaped by chronic exposure to a variety of diverse pathogens may promote low rates of cardiovascular and diabetic disease [10], [14].

Second, whether cumulative activity up through early adulthood or sustained physical activity throughout late adulthood are more important for delaying late age morbidity is an open question. Given the lack of cardiovascular and metabolic disease observed among Tsimane, we predict that men and women will maintain high levels of activity late into adulthood despite declining functional status. Men and women may, however,differ substantially in their activities, and in their subsequent chronic disease risk.

Third, the pace of economic and epidemiological change has accelerated among Tsimane since the 1970's with regional population growth and the introduction of roads, schools and a health clinic [15]. A large literature supports the notion that lifestyle changes associated with greater modernization contribute to hypertension, obesity and their sequelae, despite reduced incidence of infectious disease and potentially greater access to healthcare ([16] and refs therein). A common route by which modernization can escalate non-infectious morbidity is through changes in activity profiles and obesity. Despite higher mortality and infectious morbidity in more remote Tsimane villages, we have not observed substantial variability among Tsimane villages in their risk of hypertension, hypercholesterolemia or peripheral arterial disease [10], [17], [18]. We therefore hypothesize that physical activity profiles will not differ substantially by level of modernization, particularly because all Tsimane continue to rely heavily on a subsistence economy.

Fourth, the advent of plant and animal domestication and reduced mobility among semi-sedentary horticulturalists compared to nomadic hunter-gatherers is expected to have altered lifeways and health [9]. Disease ecology is expected to differ between hunter-gatherers and horticulturalists as well, due to differences in population density, sedentism and proximity to domesticated animals [19], which should affect levels of infection-induced inflammation and rates of physical decline and chronic disease; yet hunter-gatherers and horticulturalists display similar age-schedules of survivorship [20], [21] and fertility [22], [23]. We compare levels of Tsimane physical activity to those observed among other subsistence populations. Given the very high fertility of Tsimane women (Total Fertility Rate  =  9 births per woman), we expect that Tsimane women's activity levels will be lower than those observed among hunter-gatherers.

To address these questions, we employ a factorial method [24] based on a large representative sample of behavioral observations to estimate Tsimane physical activity levels (PALs) by age, sex, and season. We test for effects of modernization on activity level by assessing variability in PAL by Spanish fluency, formal schooling and geographical regions that vary in access to markets, wage labor and health care. We also test whether nascent overweight and obesity are associated with reduced physical activity. Whether physical activity is associated with weight loss, gain or no net effect is a controversial topic with much debate [25].

In addition, we utilize accelerometry and heart rate data on a smaller sample to evaluate evening and nighttime activity, and to corroborate the factorial method. Finally, we compare our results with PALs from other populations to situate Tsimane physical activity in the larger context of subsistence and market economies.

## Materials and Methods

### Study population

The Tsimane are lowland forager-horticulturalists in the Beni region of Bolivia with a total population of ∼11,000, living in 90+ villages comprised of extended family clusters. The Tsimane diet consists of cultivated staples from slash and burn horticulture (66%), wild game from hunting (17%), freshwater fish (7%), and fruits and nuts from gathering (6%) [26]. Market foods (e.g. sugar, pasta, crackers) and domesticated animals (e.g. chicken, pork and beef) each provide 2% of the daily calories. Many Tsimane are isolated from modern society and have not yet undergone an epidemiological and technological transition. Only a couple villages have any electricity, and there is still no running water or waste management. However, as new roads were built in the 1970s, and with greater socioeconomic development in the Beni, the Tsimane have come into greater contact with outsiders [15].

### Ethics Statement

Informed written consent was obtained for all protocols from the Gran Consejo Tsimane, the local Tsimane government organization that represents Tsimane interests and oversees all projects. Verbal consent was obtained from community officials and study participants by fingerprint due to limited literacy in the population. Consent procedures and protocols were approved by the University of California, Santa Barbara and University of New Mexico Institutional Review Boards.

### Data Collection

We measure physical activity using three methods: factorial method based on behavioral observation (instantaneous scan sampling), accelerometery, and accelerometry combined with heart rate monitoring. Our goal was to obtain three estimates of 24-hr physical activity levels (PAL), defined as the ratio of total daily energy expenditure to daily basal metabolism.

Time allocation data are instantaneous scan samples of all members of household residential clusters during two- or three-hour observation blocks, ranging from 7am to 7pm, in six villages from July 2002 through November 2005 (n = 70,574 scans on 967 individuals; 24,923 on 319 adults age 20+) [27]. During these blocks, all activities of members from several families were recorded every half hour. If two activities were performed simultaneously (e.g. holding an infant while walking to a garden), both were recorded and given equal weighting. Observations outside of the village residential cluster were mostly confirmed by other village members or by focal individuals after returning home. However, the exact activity cannot be known for non-observed activities and so specificity may be lacking using factorial method.

For accelerometry, we used an Actigraph uniaxial accelerometer with Polar Wearlink® heart monitor (Actitrainer) to measure activity and to estimate daily energy expenditure on 52 adults in a different village from Sept–Oct 2008, Jan–March and July–Aug 2009. Several adults were sampled on two non-consecutive days, resulting in 65 person-days of data. Only samples covering at least 75% of each 12 hr interval (9 hrs) were included (n = 34, 33% female). In this restricted sample, only one woman was pregnant and one woman was lactating during time of study; both are included in the analysis. Participants were instructed to wear the accelerometer on the right hip and to maintain usual habits on sample days. Data were downloaded in 10 s epochs in 24 h bouts and recoded into 1 min intervals.

### Factorial method

PALs were first estimated using a factorial method with time allocation data [24]. While the factorial method has been criticized for underestimating total daily energy expenditure (TDEE) relative to doubly labeled water (DLW) [28], our purpose here is to estimate PAL and not to measure energy expenditure directly. Each activity code has an associated physical activity ratio (PAR) that describes average intensity as a multiplier of estimated basal metabolic rate (BMR) [29]. We first calculate a daytime average PAR by sex, age group (20–39, 40–59, 60+), region (riverine, forest, near town) and season (wet, dry, intermediate). Daytime PAR was obtained by averaging PARs across individuals in each category. PARs are weighted to insure equal time point representation throughout the day and by season.

In order to calculate a 24-hr PAL using factorial-based data, we require information on activity levels during non-daytime hours. Evening and nighttime PAR (7pm–7am) was estimated as the average of the two accelerometry methods at 1.36 for women (n = 16) and 1.42 for men (n = 23). By age, the evening and nighttime PAL for both sexes combined is 1.45 (20–39 yrs, n = 21), 1.34 (40–59 yrs, n = 13), and 1.33 (60+ yrs, n = 5). These values were averaged with daytime PAR from the factorial method (Table 1) to provide estimates of 24 hr PAL. PAL values for a sedentary and light lifestyle range from 1.4 to 1.69, while a “moderate to active” lifestyle includes PALs ranging from 1.70–1.99 and a “vigorously active” lifestyle' is associated with PALs of 2.0 or greater [29].

**Table 1:** Mean PALs by age, sex, region, and season.

Mean PAL	Age, sex											
	20–39		20–39 Total	40–59		40–59 Total	60+		60+ Total	Women Total	Men Total	Total
Region, season	Women	Men		Women	Men		Women	Men				
**Forest**	1.65	2.13	2.01	1.67	2.10	1.87	1.67	1.73	1.69	1.66	2.08	1.93
**Dry**	1.66	2.45	2.24	1.69	2.40	2.02	1.75	1.80	1.77	1.68	2.38	2.12
**intermediate**	1.63	1.93	1.85	1.66	1.96	1.79	1.54	1.68	1.63	1.63	1.91	1.81
**wet**	1.65	1.77	1.78	1.65	1.83	1.73	1.62	1.66	1.63	1.65	1.78	1.74
**Neartown**	1.82	2.03	1.98	1.80	2.03	1.92	1.78	1.91	1.84	1.81	2.02	1.94
**dry**	1.80	2.04	1.97	1.74	2.05	1.90	1.70	1.79	1.74	1.78	2.01	1.92
**intermediate**	1.84	2.05	1.99	1.88	2.01	1.95	1.86	2.04	1.95	1.85	2.04	1.97
**wet**	1.83	1.77	1.85	1.85	1.99	1.92	1.98	2.06	2.01	1.85	1.85	1.87
**Riverine**	1.69	1.95	1.87	1.72	1.88	1.79	1.60	1.84	1.71	1.69	1.92	1.83
**dry**	1.66	1.88	1.82	1.64	1.88	1.76	1.71	1.89	1.78	1.66	1.88	1.79
**intermediate**	1.69	1.94	1.87	1.71	1.86	1.78	1.56	1.83	1.68	1.69	1.91	1.82
**wet**	1.71	2.02	1.91	1.80	1.92	1.85	1.56	1.84	1.73	1.72	1.99	1.87
**Total**	1.74	2.05	1.96	1.72	2.03	1.87	1.70	1.82	1.75	1.73	2.02	1.91

Note: Daytime PARs based on factorial method from Table S1 are averaged with PARs from evening and nighttime monitoring using the Accelerometer-HR method (PARn: Women  =  1.358, Men  =  1.422, 20–39 y  =  1.448, 40–59 y  =  1.336, 60+  =  1.330) (see text).

Women's reproductive status was considered in analyses of PAR and BMI. “Pregnancy” was operationalized as the period of six months prior to a birth (16% of observations among women aged 20–45) and “lactation” as the period up to one year post-birth (33% of observations).

### Accelerometry and heart rate

PARs were estimated using just an accelerometer, and by combining accelerometry with heart rate (HR). The accelerometry method used the Freedson equation [30] to estimate PAR from accelerometry counts per minute (CPM) for moderate-vigorous activity: 1.439+.000795*CPM (CPM≥1951) and the Yngve et al. equation [31] otherwise: 1.136+0.0008249*CPM (CPM<1951). Five activity levels were defined by CPM ranges in accordance with Freedson: sedentary (CPM = 0 to 99), light (100–759), lifestyle (760–1951), moderate (1952–5724), vigorous (5725+). PAL was calculated by averaging across separate daytime and evening hourly PAR estimates, to take full advantage of hours sampled.

Accelerometry largely registers walking, running and other horizontal movement, but does not adequately capture resistance-based movements where the torso does not move sufficiently, and so it is expected that accelerometry underestimates PALs when used alone [32]. For this reason, several studies have shown no relationship between TDEE or PAL and accelerometry counts [33]. The third method (Accelerometry-HR) to estimate PAL therefore assesses activity-based energy expenditure (AEE) using information from the HR monitor when the accelerometer registers low activity [34]. The Accelerometry-HR method first estimates average daily-living heart rate (ADLHR), the average heart rate when HR<80 beats per minute (bpm) and CPM>100. Average active heart rate (AAHR) is then estimated as the average heart rate when CPM>1951 and HR>79 bpm. AAHR and Freedson equations are used to estimate average active caloric expenditure (AACE). A calibration ratio was calculated by dividing AACE by ΔHR = AAHR-ADLHR. This is an estimate of energy expenditure per BPM above ADLHR. Activity-based energy expenditure (AEE) for each epoch was estimated by multiplying the calibration ratio by the bpm for each epoch when heart rate was above ADLHR and was calculated using the Freedson equation when heart rate was below ADLHR.

By accounting for low levels of activity, the Accelerometer-HR method should provide larger estimates of AEE than accelerometry alone. At least one study has shown that combining accelerometry with heart rate can provide estimates of energy expenditure for free-living activity similar to those obtained from DLW [35]. Due to variability in beginning and end times, daytime and nighttime AEE/min was calculated separately and then multiplied by 720 min to estimate AEE_Day_ and AEE_Night_ for the respective 12 hr daytime or nighttime interval. PAL is then calculated as (BMR+AEE_Day_+AEE_Night_)/BMR, where BMR is the basal metabolic rate as estimated from the Oxford equations [36].

### Modernization variables

#### Geographic region (riverine, forest, near town)

The three geographic regions represented here differ in their reliance on fishing versus hunting, access to health care, and proximity to markets and wage labor opportunities–all relevant for testing the influences of modernization on physical activity patterns. Both the riverine and forest villages are located at least a day's travel (by canoe and truck, respectively) from the closest market town, San Borja. However, in the riverine region, proximity to a Catholic mission provides some access to health care and formal education, and there is limited trade along the river. The forest region offers opportunities for wage labor with logging companies in the dry season. The near town region is a 1 hr taxi ride from San Borja. In and around San Borja, Tsimane sell cash crops, purchase clothes, visit the hospital and health clinic, and obtain wage labor opportunities usually as ranch hands.

#### Education (highest grade achieved – 0–15)

A majority of Tsimane villages currently have an elementary school (grades 1–5). Classes are typically only half day, but attendance may be sporadic, and classes often involve traditional skills such as fishing. More advanced education (grades 6–12) is only available in the near town villages.

#### Spanish fluency (none, some, fluent)

Spanish fluency, rather than education tends to be the limiting factor for access to wage labor [37]. Spanish fluency is attained through school, non-Tsimane parents, or interactions with Bolivian nationals and differs by age (younger cohorts speak better Spanish), sex (males > females), and region (near town > riverine > forest).

### Data Analysis

#### PAR

To explore individual variation in activity, we ran linear mixed-effects models (LMMs) of PAR*_i,t,n_*, where PAR*_i,t,n_* is the activity-specific physical activity ratio (PAR) from a spot observation of individual *i* at time *t* from community *n*. Due to repeated measures on individuals and the time cluster sampling technique, we model individual ID and time of day nested within individual ID as random effects. Fixed effects in the model include age, age^2^, sex, season (dry, wet, intermediate), and body mass index (BMI). The effects of modernization are estimated based on geographic region (near town is baseline) and by considering the effects of schooling and Spanish fluency. LMM's were fitted using the nlme package [38] in R 2.15.0 [39].

#### BMI

The same set of factors was used to analyze variation in BMI, with PAR replacing BMI as a predictor. This analysis is based on individual means as the unit of analysis, and so no random effects were modeled; we therefore used linear regressions in R 2.15.0 [39].

We started both analyses with a full model including all factors but no interactions (Model 1). We then used a simultaneous inclusion procedure to add all possible two-way interactions of age, sex, region, and season as well as sex*education and sex*Spanish, if they improved the AIC of the first model (Model 2).

## Results

### Activity budgets

**Figure 1:**
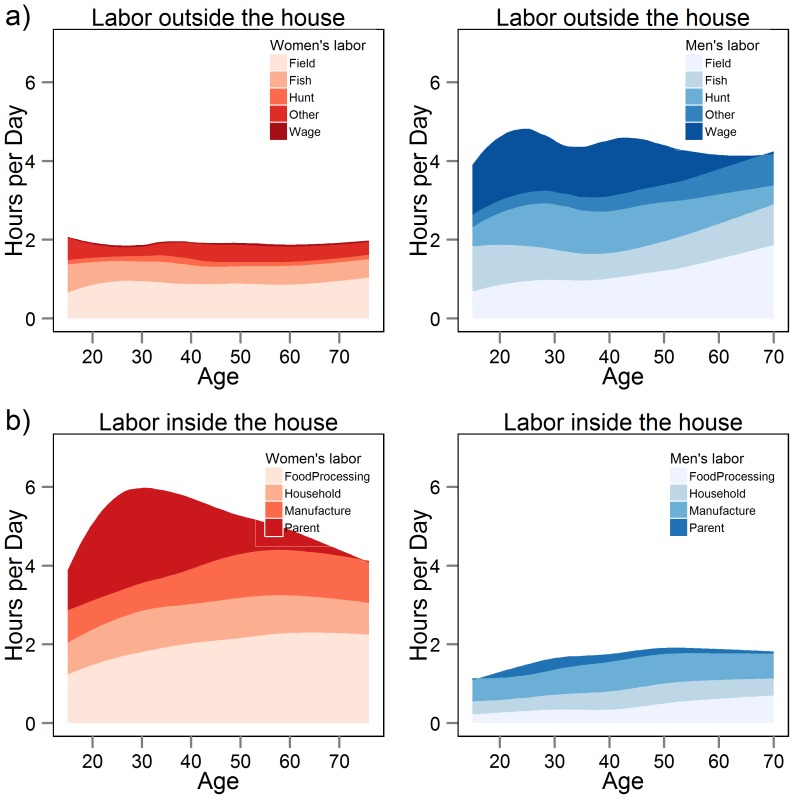
Daily time (hrs/day) men and women spend in productive labor a) outside the household and b) domestic labor inside the household, based on time allocation sampling from 7am–7pm (see text).

Time spent in subsistence and other work activities by age and sex are displayed in Figure 1. By age 15, males and females were already close to adult work levels. Men and women spent about 5 and 2 hrs/day, respectively, in direct productive tasks such as hunting, fishing and farming. Women and men spent an additional 4–6 hrs and ∼1.5 hrs/day, respectively, in domestic tasks like childcare and food processing. Direct productive tasks among women and domestic labor tasks among men remained relatively consistent over the lifespan. Among men, wage labor was concentrated among younger men, hunting between 30–45 yrs, and gardening after age 50. Among women, time spent in direct production was constant, while childcare peaked from ages 20–40 and food processing time increased throughout adulthood.

**Figure 2:**
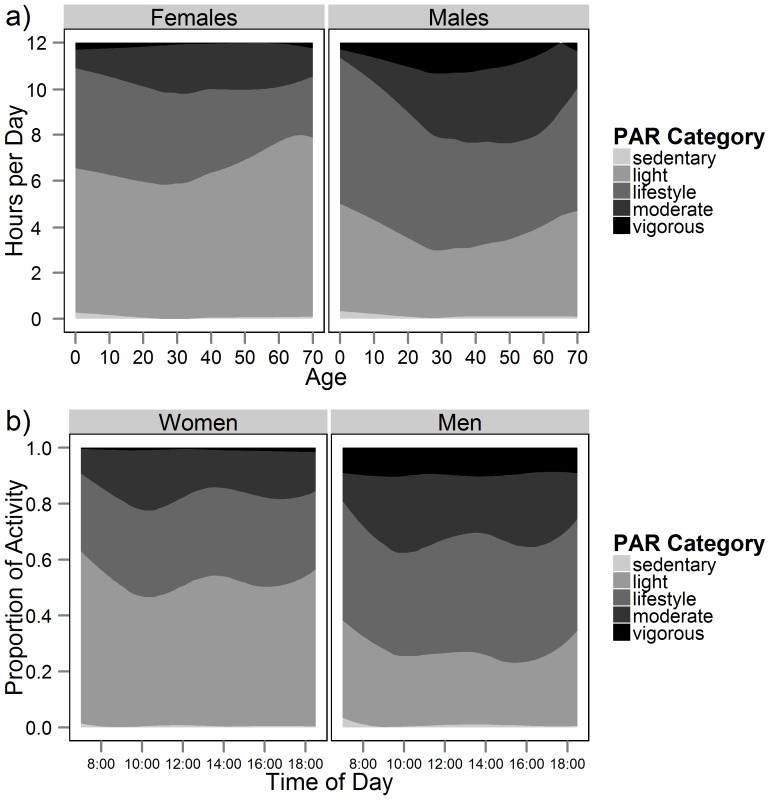
Physical activity ratios (PARs) based on factorial method clustered into categories of activity intensity. (a) Average time spent by PAR intensity for males and females (7am–7pm). (b) PAR intensity categories by time of day for adults (20+) only.

We classified daytime activity by activity category (sedentary, light, lifestyle, moderate, vigorous based on PARs) in Figure 2. Figure 2a shows about 4–6 hrs/day for women and 6–7 hrs/day for men spent in lifestyle-moderate activity; however, vigorous activity among women was minimal and represented up to 1.2 hrs/day among men. Over half of women's and about a third of men's daytime was spent in “light” activity, whereas hardly any time was spent in the “sedentary” category by men or women.

Figure 2b shows activity by time of day. Activity was lowest early in the day and late in the evening, with a slight dip in activity level in mid-day during lunchtime for both men and women. The proportion of men (or time spent) in vigorous activity was constant throughout the day, due largely to wage labor spent logging.

### PARs and PALs by age, sex region and season: factorial method

**Figure 3:**
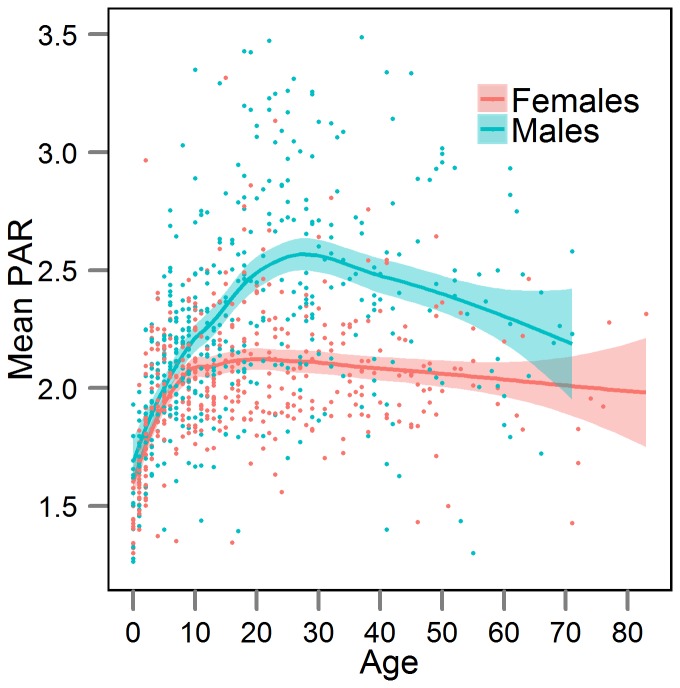
Mean physical activity ratio (PAR) by age and sex. Each data point represents one individual. PARs are derived from the factorial method, based only on observations from 7am–7pm (see text). The displayed curves are loess fits with 95% confidence intervals.

Daytime PAR (7am–7pm) for Tsimane men and women aged 20+ was 2.69 and 2.10, respectively (Table S1). Daytime PARs by age and sex are displayed in Figure 3 (and Figure S1 by region). Peak adult levels occurred by late twenties in men and by early teens in women. There was only a slight decline evident in daytime PAR with age among women and stronger declines with age among men that vary across regions (Figure 3, Figure S1). Dry season adds about 0.4 PARs to adult men activities, on average, but does not affect women's PARs (Table 2).

**Table 2:** Physical activity levels (PALs) for subsistence popuations.

			Male		Female	
Economy	Population	Method	n	PAL	n	PAL
HG	!Kung	Factorial	n/a	1.68	n/a	1.56
HG	Ache	Factorial	n/a	2.17	n/a	1.88
HG	Hadza	DLW	13	2.26	17	1.78
HG	Igloolik Eskimo	Factorial	n/a	2.20	n/a	1.80
FH	Huli (PNG)^1^	HRM	15	1.84	12	1.88
FH	Machiguenga	Factorial	60	2.14	n/a	1.67
FH	Shuar	Acc	23	1.54	26	1.42
**FH**	**Tsimane**	**Factorial**	**n/a**	**2.02**	**n/a**	**1.73**
**FH**	**Tsimane**	**Acc**	**22**	**1.47**	**14**	**1.46**
**FH**	**Tsimane**	**Acc+HRM**	**22**	**2.15**	**14**	**1.85**
AGFISH	Luo	Acc+HRM	172	1.93	209	1.81
PAST	Yakut	DLW	14	1.68	14	1.50
PAST	Evenki	Factorial	17	1.41	44	1.42
PAST	Evenki	HRM	17	1.48	44	1.59
AGPAST	Masaai	Acc+HRM	163	1.95	178	1.99
AGPAST	Bolivian Aymara, 1990^2^	DLW	6	1.96	6	2.04
AGPAST	Bolivian Aymara, 1997^3^	DLW	7	2.18	7	2.26
FARM	Kamba	Acc+HRM	94	1.95	283	1.90
FARM	Highland Ecuador	HRM	11	2.39	11	1.97
FARM	Coastal Ecuador	HRM	5	1.58	5	1.63
FARM	Farming Societies	DLW	11	2.08	14	2.11
	AVERAGE			1.91		1.77

Note: Data sources include [12], [28], [11], [67], [68] and [42]. HG = hunter-gatherer, FH = forager-horticulturalist, PAST = pastoralist; DLW = doubly-labelled water method, Acc = accelerometry, HRM = heart rate monitor; AGPAST = agropastoralist, FARM = intensive agriculturalists;

1 also engaged in pig husbandry;

2 during low work season,

3 during high work season.

Overall men's 24-hr PAL was 2.02 and women's 1.73, after including accelerometry-HR PARs for nighttime (7pm–7am) activity when behavioral observation was not done. PAL was at its peak from 20–39 among men, declining from 2.05 to 1.82 by age 60+, whereas women's PAL was constant throughout adulthood at around 1.70–1.74 (Table 1). Tsimane inhabiting forest villages appeared more active than those living on the Maniqui River (1.93 vs. 1.73, Figure S1). Contrary to expectations, Tsimane living close to town did not exhibit low PAL (1.81 for women, 2.02 for men, Figure S1). Seasonality did not affect PALs of women under 60 yrs, whereas men's PAL was highest in the dry season. Higher dry season activity among men was based largely on the sample of villages in the forest region (Table 1). The highest PAL for men was among 20–39 yr olds in forest villages during the dry season (PAL = 2.45), whereas the lowest occurred among men age 60+ in forest villages during the wet season (PAL = 1.66). The highest PAL for women was among 60+ yr olds living near town during the wet season (PAL = 1.98), whereas the lowest was among 60+ yr olds in forest villages during the season intermediate between dry and wet (PAL = 1.54).

### PALs: Accelerometry and Accelerometry-Heart Rate (HR) method

Combining heart rate with accelerometry increased the estimated PALs just above the factorial method estimates: 24-hr accelerometry-HR PALs were 2.15 for men and 1.85 for women (Table 2). Among adults with day and night adequately sampled, mean±SD PAL is 2.23±0.55 (range: 1.42–3.62, n = 18) for men and 1.82±0.31 (range: 1.45–2.53, n = 11) for women. Using only accelerometry (without heart rate), mean 24-hr PAL was 1.47 for men (n = 21) and 1.46 for women (n = 12) (Table 2), the lowest estimate of PALs of the three methods tested in this paper and significantly different than the other two methods (women: t_1_ = 5.5, p(one-tailed) = 0.06; men: t_1_ = 9.46, p = 0.03). Among adults with day and night adequately sampled, mean±SD PAL is 1.49±0.14 (range: 1.26–1.69, n = 18) for men and 1.46±0.14 (range: 1.34–1.87, n = 11) for women.

**Figure 4:**
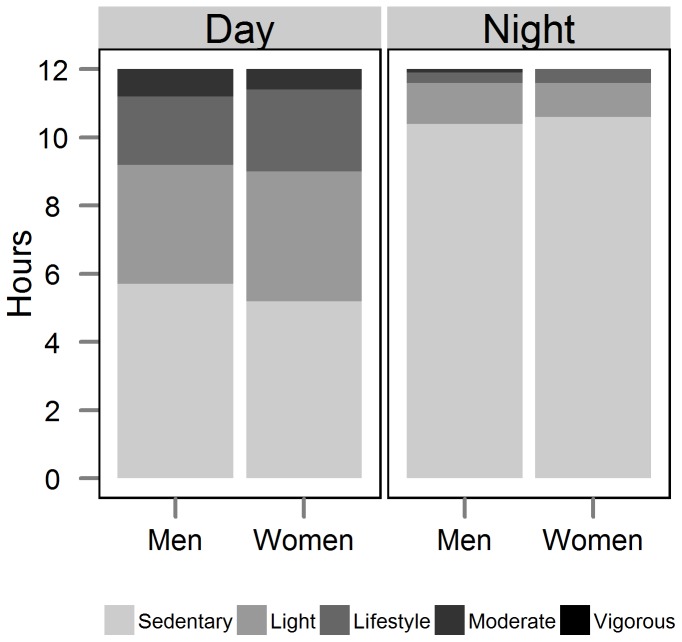
Hours per day by activity level, from accelerometry. Shown separately for men and women and for daytime (7am–7pm) and nighttime (7pm–7am) intervals. See text for definition of activity level categories.

Figure 4 displays the amount of time spent in the same five activity categories from Figure 2 (sedentary, light, lifestyle, moderate, vigorous) but using accelerometry. Consistent with the lower overall PAL from accelerometry (Table 2), we found a much larger percentage of daily time spent sedentary and in light activity by accelerometry than by factorial method. The accelerometry method did not identify any vigorous activity, with just 2–2.4 hrs/day spent in moderate activity.

### Modeling daytime physical activity (PAR)

**Figure 5:**
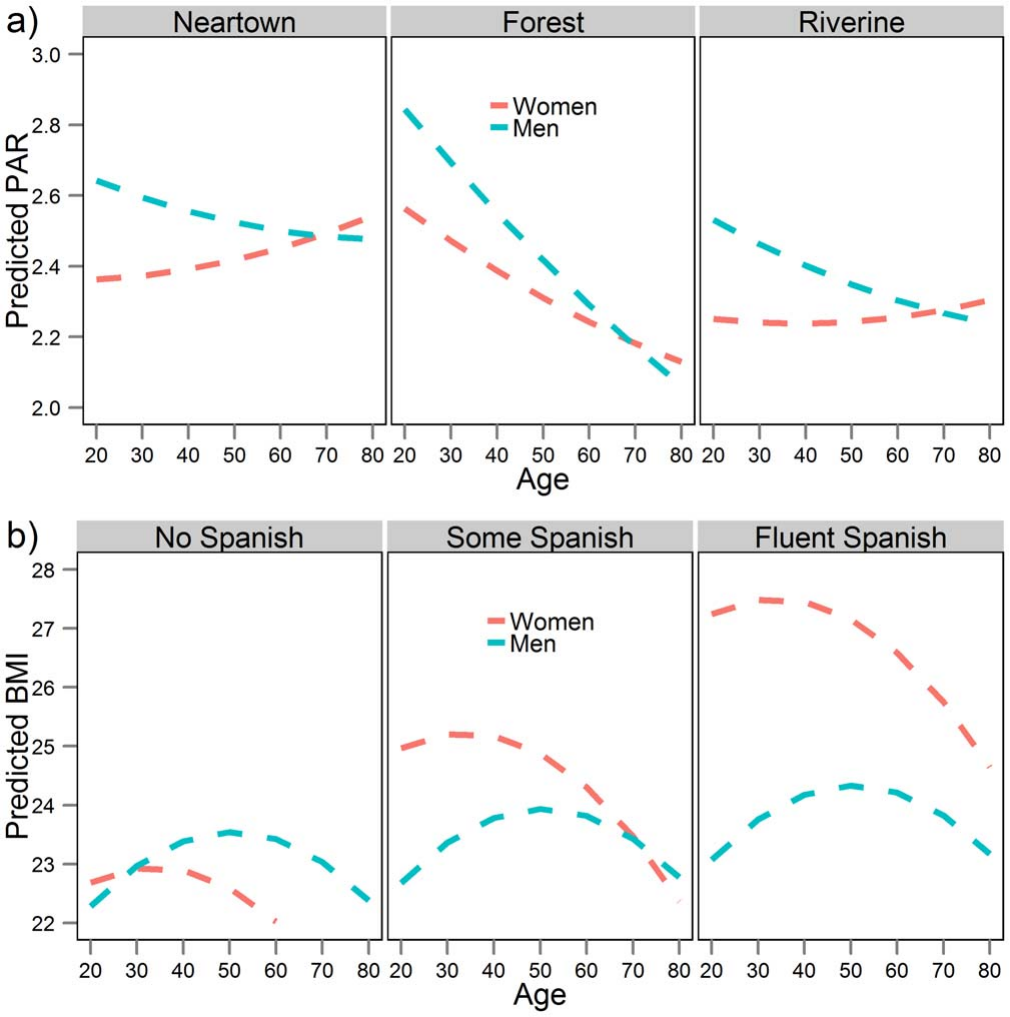
Predicted values of (a) PAR by age, sex, and region, and (b) BMI by age, sex, and Spanish fluency based on Model 2 in **Table 3** and **4**, respectively. All other variables held at baseline or population average.

We used linear mixed-models to examine the effects of age, sex, season, region, education, and Spanish fluency on the PAR of observed activities. The model permits tests of whether individual physical activity varies by demographic and modernization variables. The mixed-effects model confirms that men were more active than women (by 0.44 PAR, Table 3, Model 1). Adding interaction terms in Model 2 slightly diminishes the sex difference at late ages and shows seasonal and regional differences between men and women (Figure 5a, Table 3). Men in remote forest and riverine villages engaged in activities that were 0.52 and 0.25 PARs higher than those performed by women. Men were more active than women during the dry season (0.27 PAR), but were less active than women during the wet season (−0.36 PAR). Excluding the high PARs derived from logging activities (set conservatively at PAR = 6.6 [29]), the sex difference in PAR is cut in half, and men's PARs are no longer greater than women's in the forest or during the dry season (Table S3). Pregnant women observed lower PARs than non-pregnant, non-lactating women, with the difference in PAR increasing with maternal age (β_preg_ = 0.36, p = 0.026; β_preg*age_ = −0.02, p = 0.003, Table S4). At age 20, a pregnant woman has a PAR that is 2.2% higher than that of a non-pregnant, non-lactating woman; by age 40, PAR is lower in pregnant women by 11.2%.

**Table 3:** Linear mixed-effects models of PAR based on scan observations of adults age 20+ (n = 24,923 observations on 319 adults).

	Model 1 (AIC = 80600)			Model 2 (AIC = 79958)		
Factors	Estimate	±SE	t-value	Estimate	±SE	t-value
(Intercept)	2.57	0.30	8.45***	2.43	0.33	7.41***
Age	−0.01	0.01	−0.96	−0.01	0.01	−0.09
Age^2^	0.00	0.00	0.43	0.00	0.00	0.35
Sex (male)	0.44	0.06	7.44***	0.40	0.16	2.47*
Forest (vs. Near Town)	0.05	0.07	0.71	−0.11	0.21	−0.54
Riverine (vs. Near Town)	−0.12	0.06	−1.79°	−0.32	0.18	−1.77°
Dry Season (vs. intermediate)	0.12	0.02	6.54***	0.02	0.06	0.38
Wet Season (vs. intermediate)	−0.24	0.02	−10.37***	−0.19	0.11	−1.82°
Education (highest grade)	−0.01	0.01	−0.76	−0.01	0.01	−0.38
Spanish (0–2)	0.11	0.06	1.76°	0.10	0.06	1.55
BMI	−0.01	0.01	−0.76	−0.01	0.01	−0.51
Age*Sex (male)				−0.01	0.00	−1.52
Age*Forest				−0.01	0.00	−2.23°
Age*Riverine				−0.00	0.00	−0.47
Age*Dry Season				−0.01	0.00	−4.57***
Age*Wet Season				0.01	0.00	3.29***
Sex (male) *Forest				0.52	0.12	4.27***
Sex (male) *Riverine				0.25	0.12	2.11*
Sex (male) *Dry Season				0.27	0.04	7.16***
Sex (male) *Wet Season				−0.36	0.05	−7.67***
Forest*Dry Season				0.52	0.05	11.40***
Riverine*Dry Season				0.01	0.05	0.15
Forest*Wet Season				−0.03	0.09	−0.31
Riverine*Wet Season				0.27	0.09	2.95**

Note: Random effects include person ID and time of day nested within person ID. Model 1 includes only main effects, Model 2 includes two-way interactions that improved AIC in a simultaneous inclusion procedure.

° *p*<0.1,

* *p*<0.05,

** *p*<0.01,

*** *p*<0.001.

BMI was not a significant predictor of PAR (Table 3), nor did replacing BMI with body fat percentage, body mass, fat-free mass, or the combination of mass with body fat percentage produce significant effects (Table S2). Likewise, schooling did not significantly predict PAR for men or women. Spanish fluency was marginally significant in Model 1, with fluent adults more active (0.23 PARs) than monolingual Tsimane speakers (Table 3, Figure S2). However, after excluding observations of logging activities, the effect of Spanish language ability disappears (Table S3).

### Modeling BMI

The prevalence of obesity in men and women is 1.2% (n = 2) and 4.6% (n = 7), respectively. Although obesity is rare, the prevalence of overweight (25≤BMI<30) is 15.0% (n = 25) and 21.1% (n = 32), respectively. Although body size had no effects on physical activity, we considered whether physical activity predicted BMI (Table 4). Average PAR did not significantly predict BMI (Table 4), nor did it predict fat-free mass, body fat percentage, or body weight (Table S5). Women's BMI declines by age 60, whereas men's BMI increases over the same period. Women have higher BMI than men throughout much of adulthood until about age 40 (Figure 5b). Pregnancy and lactation are associated with greater BMI, independently of age and other controls, although the effects are muted at later maternal ages (Table S3). For example, a pregnant 20 year old woman has a BMI of 25.0 kg/m^2^ (10.5% higher than that of a non-pregnant, non-lactating 20 year old), whereas 35 year old women had similar BMIs regardless of reproductive status (23.8, 23.5, 23.6 kg/m^2^ for pregnant, lactating and non-pregnant/non-lactating women, respectively, Table S4).

**Table 4:** Linear regression model of BMI on 319 adults aged 20+.

	Model 1 (AIC = 1493.7)			Model 2 (AIC = 1482.3)		
Variable	Estimate	±SE	t-value	Estimate	SE	t-value
(Intercept)	20.90	1.39	15.00***	22.16	2.48	8.95***
PAR	−0.08	0.30	−0.27	−0.26	0.89	−0.29
Age (yrs)	0.13	0.06	2.40*	0.07	0.09	0.83
Age^2^	−0.00	0.00	−2.51*	−0.00	0.00	−2.06*
Sex (male)	−0.79	0.36	−2.18*	−1.66	1.14	−1.45
Forest	−0.40	0.38	−1.04	−0.35	0.38	−0.91
Riverine	−0.89	0.35	−2.57**	−0.81	0.34	−2.37*
Education (highest grade)	0.02	0.07	0.31	−0.09	0.14	−0.63
Spanish (0–2)	1.17	0.34	3.40***	2.28	0.49	4.61***
PAR*Age				0.01	0.02	0.36
Age * Sex (male)				0.05	0.02	1.84°
Sex (male)*Education				0.20	0.16	1.30
Sex (male)*Spanish				−1.89	0.66	−2.86**

° *p*<0.1,

* *p*<0.05,

** *p*<0.01,

*** *p*<0.001.

In terms of regional differences, BMI was significantly lower in the riverine communities. BMI did not vary by education but was significantly and strongly predicted by Spanish fluency. Thus, being fluent in Spanish was associated with a BMI 2.34 kg/m^2^ greater than for a monolingual Tsimane (Model 1). In addition, Model 2 reveals sex differences in this effect: Spanish fluency interacted with sex such that a fluent Spanish speaking man had a BMI that was only 0.79 kg/m^2^ greater than a monolingual man, whereas the equivalent difference for a fluent Spanish speaking woman was 4.56 kg/m^2^ (Figure 5b). The average BMI of fluent Spanish speaking women places them in the overweight category. Six of the 10 fluent women in our sample are overweight and only one has a BMI lower than 23. To confirm that effects on BMI were effects on body fat, and not muscle mass, we ran models with body fat and fat-free mass as dependent variables (Table S5). We found that associations differ by sex: Spanish fluency is associated with greater body fat percentage in women, and greater fat-free mass in men.

### Comparison with subsistence populations

Table 2 summarizes PALs for adults among other subsistence populations, including hunter-gatherers, horticulturalists, herders and intensive agriculturalists. Although PALs listed in Table 2 have been estimated from a variety of methods, the Tsimane PALs we report here are typical of other subsistence populations. Tsimane PALs were significantly higher than those of pastoralists (single sample t-test; Men_Fact_ t_2_ = −6.14, P<0.05, Men_Acc-HR_ t_2_ = −7.75, P<0.05, Women_Fact_ t_2_ = −4.62, P<0.05, Women_Acc-HR_ t_2_ = −7.06, P<0.05), but were not significantly different than PALs of hunter-gatherers (Men_Fact_ t_3_ = 0.43, P = 0.70, Men_Acc-HR_ t_3_ = −0.54, P = 0.62, Women_Fact_ t_3_ = 0.37, P = 0.74, Women_Acc-HR_ t_3_ = −1.39, P = 0.26), nor agropastoralists and farmers (Men_Fact_ t_7_ = −0.21, P = 0.84, Men_Acc-HR_ t_7_ = −1.79, P = 0.12, Women_Fact_ t_7_ = 3.46, P<0.05, Women_Acc-HR_ t_7_ = 1.69, P = 0.14) with the exception of women using the factorial method, who had significantly lower PALs.

### Comparison with industrialized populations

**Figure 6:**
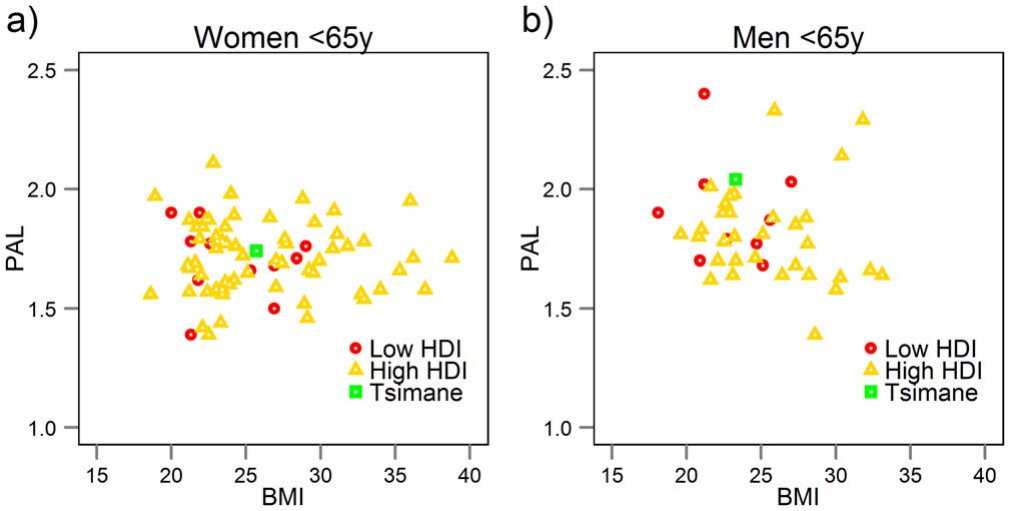
Physical activity levels (PALs) from a compendium of populations, shown separately for developing (low or middle Human Development Index (HDI) populations) and developed societies (high HDI) [**40**]. Tsimane are represented as the green triangle. Mean PAL for developing societies is 1.88 (men) and 1.70 (women), for developed societies is 1.79 (men) and 1.71 (women).

Figure 6 displays PALs from a large sample of developing and developed societies based on a recently published meta-analysis [40]. Societies were labeled as “developed” if their United Nations Human Development Index (HDI) was high, and “developing” if their HDI was low or middle. Compared to these samples, Tsimane had high PAL and slightly below average BMI. Men's PAL ranked higher than 7/9 (78%) low HDI samples and 29/32 (91%) high HDI samples. Their BMI was lower than 4/9 (44%) low and 17/32 (53%) high HDI samples, respectively. Women's PAL ranked higher than 9/11 (82%) low or middle HDI samples and 47/66 (71%) high HDI samples, while their BMI was lower than 4/11 (36%) low and 44/66 (67%) high HDI samples, respectively. While Tsimane were more physically active than many industrialized populations, their level of activity was not outside the range of existing variation in either low or high HDI context.

## Discussion

Based on factorial method and accelerometry combined with heart rate, we found that PALs among Tsimane men and women are about 0.1–0.3 units higher than those of industrialized populations, but similar to those of other subsistence-oriented populations. A difference of only 0.1 PAL might seem small, but for a typical Tsimane man (62 kg) or woman (56 kg) amounts to an additional 144 and 120 calories expended per day in activity, respectively. Despite some seasonality in production tasks, physical activity remains high throughout the year. Tsimane men show higher PALs than women at all ages and in different geographic regions of their territory; multiple regression analysis, however, showed that women display greater activity than men in the wet season when rice is harvested, but in general, men's total activity is more seasonal than women's. Men are most active during the dry season months from May to August, especially in the forest region when hunting and logging activities are common. At the end of the dry season, male work effort also intensifies during field clearance of large trees with axes and underbush with machetes.

Factorial method and accelerometry-HR methods yielded similar estimates of PAL at the population level, whereas accelerometry alone gave significantly lower PAL estimates. Other comparisons of physical activity show a similar pattern where accelerometry alone underestimates free-living activity. For example, a recent study of activity among Shuar forager-horticulturalists of Ecuador using an Actical accelerometer reported low mean PALs of 1.54 for men and 1.42 for women [12]. A PAL of 1.4 is close to the minimum measured for healthy humans in affluent societies confined to bed-rest or respiratory chambers, and so is likely an underestimate for healthy Tsimane [41]. Our study and other recent studies [42], [43] therefore suggest that accelerometry-HR is a relatively cheap, easy, and field-friendly approach for more accurately measuring activity.

Despite greater physical activity than U.S. adults, evidence of extensive vigorous activity among Tsimane was scant, especially among women. Most physical activity instead ranges from lifestyle to moderate level, with relatively little time spent “sedentary”. Similar patterns were suggested by studies among Gambian farmers [44], Aymara agropastoralists [45], and subsistence societies more generally [46]. Our results are consistent with the growing body of evidence that shows many benefits of exercise at relatively low to moderate intensity [47], and with assertions that among hunter-gatherers, the diversity of activities performed are of moderate and not vigorous intensity [8]. These activity profiles nonetheless exceed the activity recommendations by the CDC, which advocate a mix of 150 minutes per week of moderate and vigorous aerobic activity combined with muscle-strengthening activity for at least two days per week, and those of the American Heart Association and the American College of Sports Medicine recommending only 30 minutes of moderate activity at least five days per week to “promote and maintain health”. Our results showing lower rates of sedentary behavior are also instructive, in light of growing evidence showing separable effects of time spent sedentary and average energy expenditure on weight gain, metabolism and cardiorespiratory fitness (e.g. active couch potato or weekend warrior syndrome) [48].

The effects of modernization on activity levels in a subsistence economy are modest. Activity among adults living near town is no different than those living in remote villages along the Maniqui River. Adults living in remote forest villages show the highest activity levels, although much of this difference is due to logging-related wage labor, which is restricted to men. Hunting among men is also more common in forest villages, whereas fishing (less physically intensive) is more common in riverine villages. Schooling was unrelated to activity patterns, while Spanish fluency was positively associated with greater activity. Spanish fluency is associated with greater wages among Tsimane, whereas schooling does not necessarily lead to fluency nor employment opportunities [37]. Wage opportunities for Tsimane primarily include working as ranch hands, collecting and weaving palm thatch panels, cash cropping and working for logging companies. Each of these involves extensive activity; furthermore, transport to town is often done by bicycle, walking and poling dugout canoes. Cash cropping of rice and corn also involves the clearing and weeding of larger fields. Except for cash cropping and palm thatch manufacture, most wage labor opportunities are currently restricted to men.

Modernization may also not have a substantial net effect on Tsimane PALs closer to town because Tsimane in these villages still fish and farm, sometimes hunt, play soccer, visit other villages by foot or canoe. Cash cropping is also more common close to town, because of the relative ease by which Tsimane can transport their goods to market. Interaction with the market over the past half century since roads were built, however, has been mostly sporadic, rather than intense and sustained, and has not yet led to any secular changes in height [49]. Electricity has existed since 2010, but only in a couple of villages, and so television is scarce even in the most acculturated Tsimane villages.

Despite minimal effects of modernization on activity, we found significant effects of Spanish fluency (but not education) on adult BMI, particularly among women, even though overall adult obesity prevalence was low (<3%). Spanish speakers are more likely to earn wages, and therefore may have a more energy-dense diet [37]; in our sample, fluent Spanish speakers were more likely to eat market-purchased foods than non-Spanish speakers (5.4% vs.3.3%, p<0.01, t = −3.15, df = 2192.2, t-test). These items include sugar, cooking oil, bread, beef jerky (*charqui*) and pasta. A separate Tsimane study similarly found that a human capital index was associated with greater adult BMI and body fat percentage, but that the magnitudes of the relationships were small [50]. These patterns in the Bolivian Amazon contrasts with those observed elsewhere, where in the span of only a few decades, intense market integration has led to changes in body size, activity, and risk factors for chronic disease [16], [51], [52], [53].

Men's PAL is greater than women's PAL in 11/15 of the subsistence societies listed in Table 2, including the Tsimane. Among Tsimane, the sex difference in male and female physical activity levels narrow considerably by age 70. Activity reaches a peak by mid-teens for females and by late twenties in males. Even though older men participate in a wide range of subsistence tasks (Figure 1), their overall physical activity level declines by about 10–20% from the peak (Table 1; Figure 3); older women's physical activity level remains constant throughout adulthood. Among those living near town, there was no evidence of age-related declines in daily activity among those age 60+ (Table S1). One of the few other studies to examine changes in activity profiles over the lifespan in the Netherlands observed age-declines only after age 52 [54]. The rate of decline in PALs among Dutch adults was 0.060 per decade for men and 0.023 per decade for women (adapted from Table 1 from 32). The equivalent estimate for Tsimane (0.074 per decade for men, 0.030 per decade for women) reveals a similar rate of decline. This suggests that while Tsimane may remain reasonably active for much of their adult lives, they decrease the intensity of work effort with age. Activity at late ages likely declines as a result of diminishing strength, endurance, and functional and health status with age. Such a pattern suggests that late age activity is not by itself responsible for the minimal obesity, cardiovascular disease and diabetes documented among Tsimane.

BMI and other measures of body size were unrelated to physical activity in this study. This result is relevant in light of the current controversy over the relationship between activity and obesity. One prominent explanation for the obesity “epidemic” in the U.S., and increasingly throughout the world, is a reduction in physical activity throughout adulthood. The prevalence of obesity in the U.S. increased from 5% to 22% over the period of 1980 to 2005 [55]. Since 1960, the mean daily energy expenditure due to work-related activity is estimated to have dropped by over 100 calories per day for both men and women, due to the changing nature of occupations, and work-related transportation [56]. The increase in adult weight over the same time period matches this decrease in energy expenditure, suggesting that changes in activity patterns may help explain the obesity epidemic, at least in the U.S. However, leisure-related activity may have increased over the same time period, thereby compensating for the decline in work-related activity. Indeed, there is no evidence for a decline in PAL over the same 25-yr period where obesity prevalence in the U.S. quadrupled [55].

Other evidence suggests that overconsumption of food may be more to blame for the upsurge in obesity than diminished energy expenditure. First, several studies have shown that obese individuals expend a similar amount of energy as thinner individuals and that activity levels do not consistently predict weight gain [25], [57], [58]; though possessing lower PALs, movement costs among obese individuals are higher due to greater BMR, and so their active energy expenditure can resemble that of more active, but leaner individuals. Cross-cultural data on TDEE and PAL (used in Figure 6) also shows no statistically significant difference by low or high development (based on Human Development Index) [40]. Second, Hadza hunter-gatherers of Tanzania show similar total daily energy expenditure as Westerners despite displaying higher PALs, and their body fat percentage was unrelated to PAL or energetic expenditure [11]. Body fat percentage has been shown to be unrelated to PAL and energy expenditure in Western populations as well [11], [54]. Third, total daily energy expenditure of many human populations, including “sedentary” Westerners and people in developing countries, falls along the same regression line determined by body mass and ambient temperature, as 90 species of wild terrestrial mammals who are not obese [55], [59]. Such results support the claim above that TDEE among Westerners may not be unique nor is it too low. Lastly, evidence tracks a temporal relationship between changes in food supply at the population level (as proxy for food intake after adjusting for food wastage) and weight gain patterns over the past several decades [60], [61], [62]. Together these arguments lend support to the notion that food intake may be a stronger candidate than diminished activity for the recent upsurge in obesity. Our results do not support claims that obesity is associated with reduced activity. Active Tsimane with BMI>25 may instead be “fit and fat” [63].

### Limitations

The main limitation of this study is its reliance on time allocation data and accelerometry with heart rate monitor to measure AEE and PAL, instead of the more precise and accurate doubly labeled water (DLW) method. However, DLW is expensive, and measures expenditure over a limited time period of up to two weeks. Despite not using DLW, the combined accelerometry-HR method provides the best alternative approach to measuring activity [43], [64]. The factorial method is an old approach to estimating activity and expenditure. Our factorial method presented here, however, does not suffer from standard shortcomings; it was not based on interview or recall, but instead used a large number of scan observations among 900+ individuals, which permitted year-long sampling. The main drawback of our factorial method is that it does not permit continuous observation of individuals throughout the day, and it relies on published tables to assign PARs to activities.

An additional limitation is that modernization among the Tsimane is still relatively modest and has not resulted in great changes in lifestyle or subsistence patterns as the majority of Tsimane still maintain fields for horticulture, engage in hunting and fishing activities, and wage labor opportunities are largely restricted to physical work such as logging or ranching. Thus, the Tsimane may not represent an ideal test of the hypothesis that increasing modernization leads to reduced physical activity, higher obesity, and the associated health risks.

## Conclusion

A hallmark of the evolved human life history is the ability to efficiently maximize rates of nutrient-dense caloric extraction from the environment with minimal time and effort, whether as hunter-gatherers, horticulturalists or as urban dwellers working desk jobs. The ability to obtain unlimited access to rich calories at low economic and *physical* cost in modern environments means that deliberate effort may be required to curtail food intake and to increase activity. Industrialization and automation of manual labor have diminished physical activity in most occupations such that much physical activity in the 20^th^ century and beyond comes from sports and leisure. An exclusive focus on activity without simultaneous consideration of food intake is therefore unlikely to resolve the current obesity crisis [65].

However, physical activity is nonetheless a key target for health initiatives given its important positive effects on cardiovascular health, metabolism and a variety of other diseases [61], [62]. Apart from any potential effects on body size, physical activity reduces oxidative load in muscle, levels of inflammatory cytokines, blood pressure, macrophage-rich fat and improves insulin sensitivity [3]. It also associates with a favorable CVD risk profile independently of leanness [2]. Even Sumo wrestlers, despite intentional obesity (BMI>35; >25% body fat), have normal blood lipid levels during training periods, but then suffer from premature morbidity and mortality after retiring in their mid-30's [66]. Despite socioeconomic changes among Tsimane over the past five decades, relatively high levels of light to moderate activity may help protect Tsimane from a variety of chronic diseases, even if overweight and obesity prevalence begins to rise.

## Supporting Information

Figure S1 **Mean physical activity ratio (PAR) by age, sex, and region.** The displayed curves are loess fits through individual means of the raw data, with 95% confidence intervals.
(TIFF)

Figure S2 **Predicted PAR by Spanish fluency and highest grade of education.** Based on the equations of Table 4-Model 2, holding all other variables at population average or baseline.
(TIFF)

Table S1 Mean daytime PAR (7am–7pm) by age, sex, region and season, based on factorial method (see text). Estimates are corrected for time block sampling.
(DOCX)

Table S2 Linear mixed models (LMM) of daytime physical activity ratios (PAR) using measures of body size that are different from body mass index (BMI) (cf. Table 3). Other body size measures include fat-free mass (kg), body fat (%) and weight (kg).
(DOCX)

Table S3 Same as Table 3 but sample excludes observations of logging activities.
(DOCX)

Table S4 Regression models of daytime (a) physical activity ratios (PAR) and (b) BMI among women age 20–45. Linear mixed-model (LMM) of PAR uses behavioral observations as unit of analysis and includes random effects for individual and time sampled (cf. Tables 3). Ordinary least squares (OLS) regression of BMI uses individual means as unit of analysis (cf. Table 4), with reproductive state reflecting pregnancy or lactation status during time of anthropometric measurement. Controls include age, region and Spanish fluency. Baseline for region is “Near Town” and for reproductive state is “non-pregnant, non-lactating”.
(DOCX)

Table S5 Predictors of body size. Same as Table 4, Model 2, but replaces BMI with other body size variables.
(DOCX)
