# Supplementary figures and images for: Physical Activity and Modernization among Bolivian Amerindians
Source: PLoS One. 2013 Jan 31;8(1):e55679. doi: 10.1371/journal.pone.0055679 (PMC3561330; doi:10.1371/journal.pone.0055679)

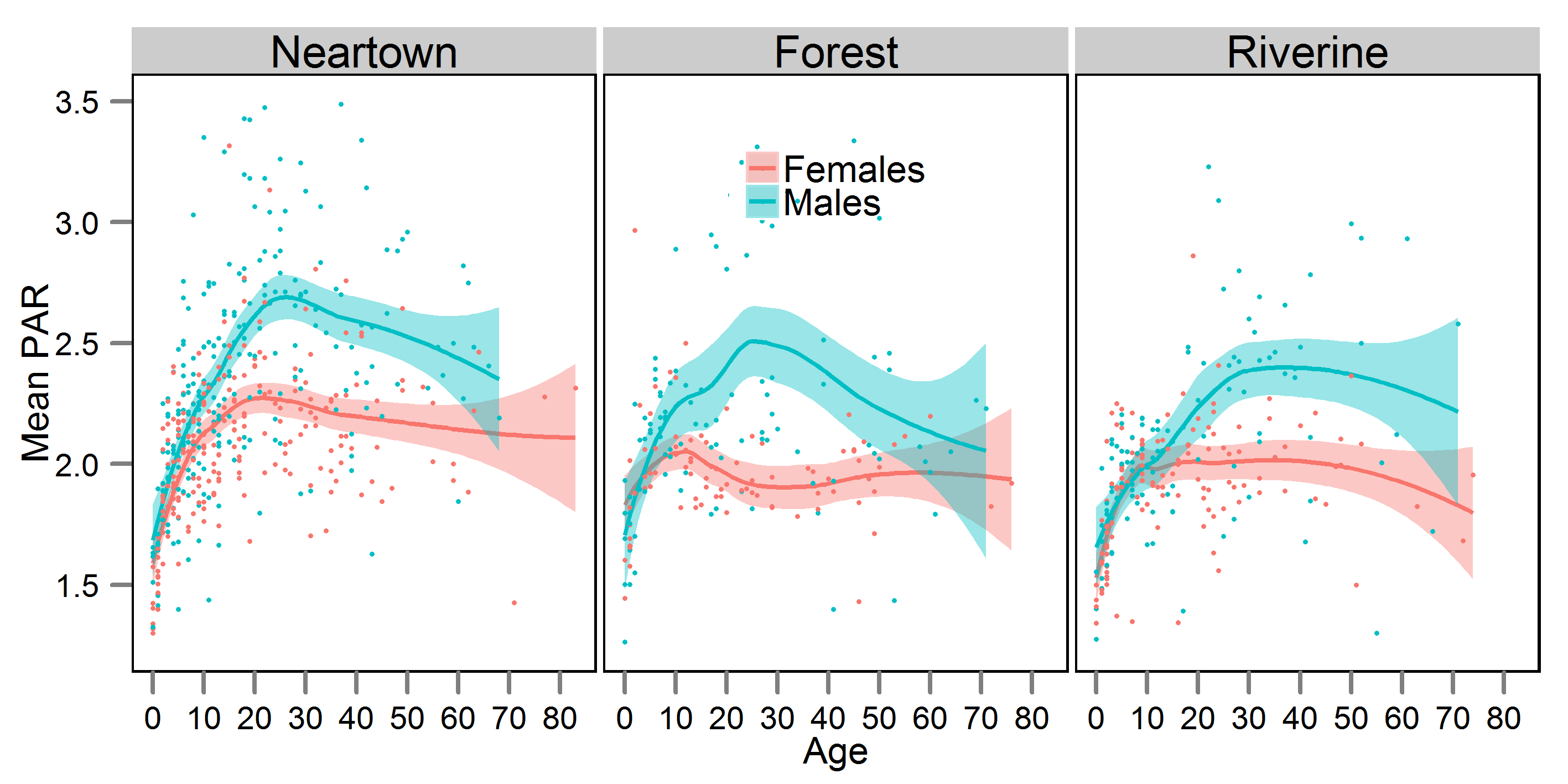

Supplement: Figure S1 — Mean physical activity ratio (PAR) by age, sex, and region. The displayed curves are loess fits through individual means of the raw data, with 95% confidence intervals. (TIFF) [file pone.0055679.s001.tiff]

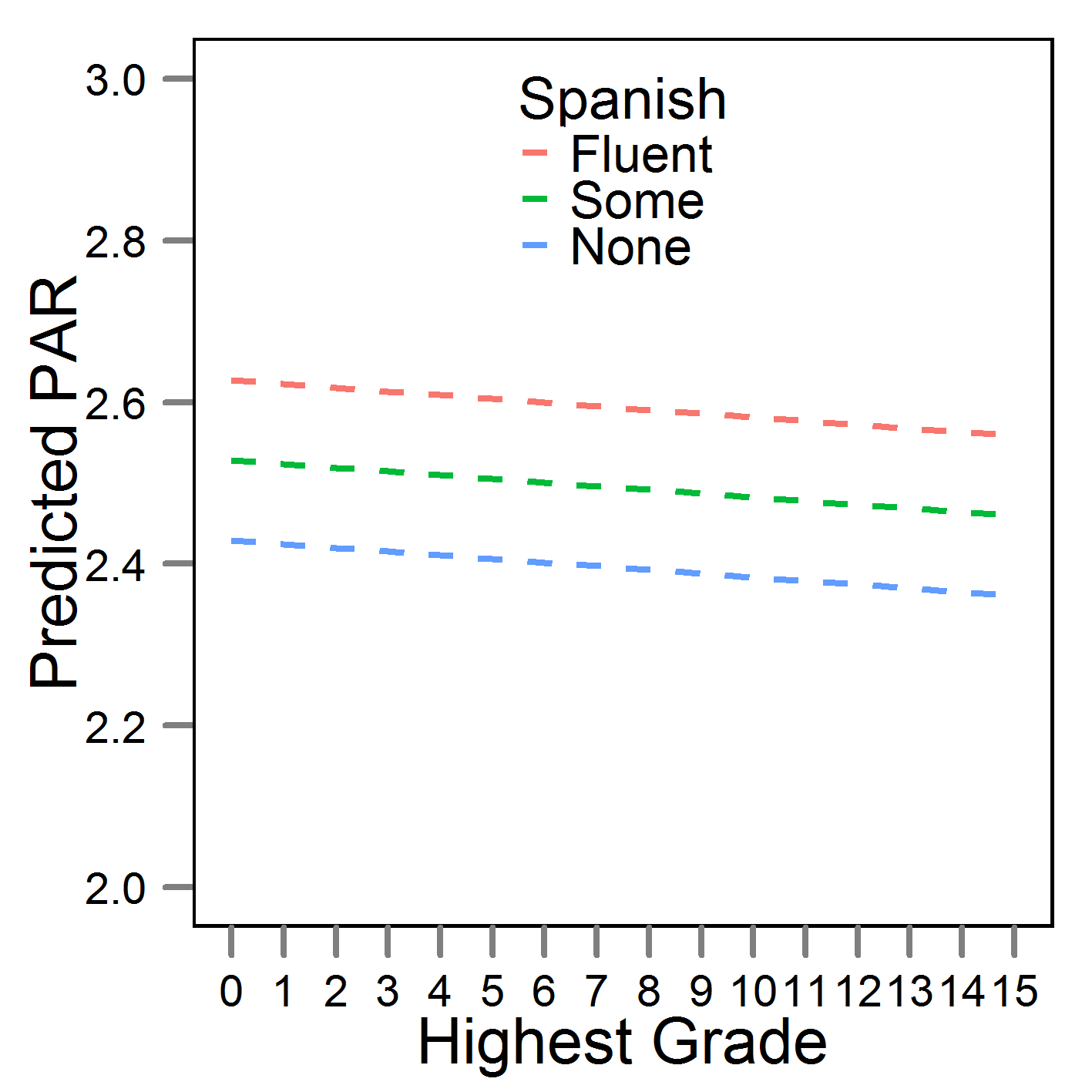

Supplement: Figure S2 — Predicted PAR by Spanish fluency and highest grade of education. Based on the equations of Table 4-Model 2, holding all other variables at population average or baseline. (TIFF) [file pone.0055679.s002.tiff]
