# Supplementary material for: Physical Activity and Modernization among Bolivian Amerindians
Source: PLoS One. 2013 Jan 31;8(1):e55679. doi: 10.1371/journal.pone.0055679 (PMC3561330; doi:10.1371/journal.pone.0055679)
Supplement: Table S1 — Mean daytime PAR (7am–7pm) by age, sex, region and season, based on factorial method (see text). Estimates are corrected for time block sampling. (DOCX) [file pone.0055679.s003.docx]

**SUPPLEMENTARY TABLE S1.** Mean daytime PAR (7am-7pm) by age, sex, region and season, based on factorial method (see text). Estimates are corrected for time block sampling.

|  | **0-19** | | **0-19 Total** | **20-39** | | **20-39 Total** | **40-59** | | **40-59 Total** | **60+** | | **60+ Total** | **Total** |
| --- | --- | --- | --- | --- | --- | --- | --- | --- | --- | --- | --- | --- | --- |
| **Region, Season** | **Girls** | **Boys** |  | **Women** | **Men** |  | **Women** | **Men** |  | **Women** | **Men** |  |  |
| **Forest** | 1.99 | 2.15 | 2.08 | 1.95 | 2.90 | 2.57 | 1.98 | 2.84 | 2.40 | 1.98 | 2.09 | 2.05 | 2.26 |
| **Dry** | 2.05 | 2.24 | 2.16 | 1.97 | 3.54 | 3.03 | 2.03 | 3.44 | 2.71 | 2.13 | 2.25 | 2.20 | 2.48 |
| **intermediate** | 1.90 | 2.11 | 2.02 | 1.89 | 2.50 | 2.26 | 1.96 | 2.56 | 2.24 | 1.73 | 2.00 | 1.93 | 2.11 |
| **Wet** | 1.96 | 2.04 | 2.01 | 1.94 | 2.19 | 2.10 | 1.94 | 2.30 | 2.12 | 1.88 | 1.96 | 1.93 | 2.05 |
| **Neartown** | 2.01 | 2.22 | 2.12 | 2.28 | 2.71 | 2.50 | 2.24 | 2.70 | 2.51 | 2.20 | 2.45 | 2.35 | 2.25 |
| **Dry** | 2.03 | 2.23 | 2.14 | 2.24 | 2.71 | 2.49 | 2.12 | 2.73 | 2.47 | 2.04 | 2.23 | 2.15 | 2.25 |
| **intermediate** | 2.00 | 2.21 | 2.12 | 2.31 | 2.74 | 2.53 | 2.40 | 2.66 | 2.56 | 2.36 | 2.72 | 2.56 | 2.26 |
| **Wet** | 1.80 | 1.98 | 1.90 | 2.31 | 2.18 | 2.25 | 2.33 | 2.63 | 2.51 | 2.61 | 2.77 | 2.69 | 2.05 |
| **Riverine** | 1.88 | 1.99 | 1.93 | 2.02 | 2.54 | 2.29 | 2.07 | 2.40 | 2.25 | 1.84 | 2.33 | 2.10 | 2.05 |
| **Dry** | 1.84 | 1.94 | 1.89 | 1.96 | 2.39 | 2.18 | 1.91 | 2.40 | 2.18 | 2.06 | 2.42 | 2.22 | 2.00 |
| **intermediate** | 1.86 | 1.92 | 1.89 | 2.03 | 2.52 | 2.29 | 2.07 | 2.36 | 2.22 | 1.76 | 2.29 | 2.02 | 2.02 |
| **Wet** | 1.95 | 2.14 | 2.04 | 2.06 | 2.69 | 2.37 | 2.25 | 2.48 | 2.37 | 1.76 | 2.32 | 2.12 | 2.15 |
| **Total** | 1.97 | 2.15 | 2.06 | 2.12 | 2.74 | 2.47 | 2.08 | 2.70 | 2.41 | 2.03 | 2.28 | 2.18 | 2.20 |
