# Supplementary material for: Physical Activity and Modernization among Bolivian Amerindians
Source: PLoS One. 2013 Jan 31;8(1):e55679. doi: 10.1371/journal.pone.0055679 (PMC3561330; doi:10.1371/journal.pone.0055679)
Supplement: Table S2 — Linear mixed models (LMM) of daytime physical activity ratios (PAR) using measures of body size that are different from body mass index (BMI) (cf. Table 3). Other body size measures include fat-free mass (kg), body fat (%) and weight (kg). (DOCX) [file pone.0055679.s004.docx]

**SUPPLEMENTARY TABLE S2.** Linear mixed models (LMM) of daytime physical activity ratios (PAR) using measures of body size that are different from body mass index (BMI) (cf. Table 3). Other body size measures include fat-free mass (kg), body fat (%) and weight (kg).

|  | Fat-free Model 1 | | | Fat-free Model 2 | | | Fat Model 1 | | | Fat Model 2 | | |
| --- | --- | --- | --- | --- | --- | --- | --- | --- | --- | --- | --- | --- |
| **Factors** | **Estimate** | **±SE** | **t-value** | **Estimate** | **±SE** | **t-value** | **Estimate** | **±SE** | **t-value** | **Estimate** | **±SE** | **t-value** |
| (Intercept) | 2.11 | 0.30 | 7.04*** | 2.16 | 0.31 | 6.91*** | 2.47 | 0.23 | 10.72*** | 2.65 | 0.23 | 11.45*** |
| Age | -0.01 | 0.01 | -1.21 | 0.00 | 0.01 | -0.47 | -0.01 | 0.01 | -0.90 | -0.01 | 0.01 | -0.98 |
| Age^2^ | 0.00 | 0.00 | 0.74 | 0.00 | 0.00 | 0.47 | 0.00 | 0.00 | 0.42 | 0.00 | 0.00 | 0.60 |
| Sex | 0.37 | 0.08 | 4.53*** | 0.42 | 0.16 | 2.56* | 0.41 | 0.07 | 5.68*** | 0.20 | 0.09 | 2.20* |
| Forest | 0.06 | 0.07 | 0.88 | -0.51 | 0.10 | -5.02*** | 0.06 | 0.07 | 0.77 | -0.50 | 0.10 | -4.90*** |
| Riverine | -0.09 | 0.07 | -1.45 | -0.39 | 0.09 | -4.18*** | -0.11 | 0.06 | -1.74° | -0.41 | 0.09 | -4.37*** |
| Dry Season | 0.13 | 0.02 | 6.88*** | 0.04 | 0.06 | 0.61 | 0.13 | 0.02 | 6.91*** | 0.04 | 0.06 | 0.66 |
| Wet Season | -0.24 | 0.02 | -10.37*** | -0.19 | 0.11 | -1.77° | -0.24 | 0.02 | -10.37*** | -0.18 | 0.11 | -1.73° |
| Education | -0.01 | 0.01 | -0.92 | -0.01 | 0.01 | -0.69 | -0.01 | 0.01 | -0.81 | -0.01 | 0.01 | -0.54 |
| Spanish | 0.09 | 0.07 | 1.36 | 0.05 | 0.07 | 0.79 | 0.11 | 0.07 | 1.63 | 0.08 | 0.07 | 1.19 |
| **Fat-free mass** | **0.01** | **0.01** | **1.38** | **0.01** | **0.01** | **1.30** |  |  |  |  |  |  |
| **% Body Fat** |  |  |  |  |  |  | **0.00** | **0.01** | **-0.78** | **0.00** | **0.01** | **-0.36** |
| Age*Sex |  |  |  | -0.01 | 0.00 | -1.99* |  |  |  |  |  |  |
| Age*Dry Season |  |  |  | -0.01 | 0.00 | -4.56*** |  |  |  | -0.01 | 0.00 | -4.57*** |
| Age*Wet Season |  |  |  | 0.01 | 0.00 | 3.25** |  |  |  | 0.01 | 0.00 | 3.21** |
| Sex*Forest |  |  |  | 0.52 | 0.12 | 4.18*** |  |  |  | 0.51 | 0.12 | 4.06*** |
| Sex*Riverine |  |  |  | 0.26 | 0.12 | 2.15* |  |  |  | 0.26 | 0.12 | 2.16* |
| Sex*Dry Season |  |  |  | 0.26 | 0.04 | 6.93*** |  |  |  | 0.26 | 0.04 | 6.86*** |
| Sex*Wet Season |  |  |  | -0.35 | 0.05 | -7.46*** |  |  |  | -0.35 | 0.05 | -7.54*** |
| Forest*Dry Season |  |  |  | 0.52 | 0.05 | 11.25*** |  |  |  | 0.52 | 0.05 | 11.23*** |
| Riverine*Dry Season |  |  |  | 0.00 | 0.05 | 0.02 |  |  |  | 0.00 | 0.05 | 0.01 |
| Forest*Wet Season |  |  |  | -0.03 | 0.09 | -0.36 |  |  |  | -0.03 | 0.09 | -0.36 |
| Riverine*Wet Season |  |  |  | 0.25 | 0.09 | 2.77** |  |  |  | 0.25 | 0.09 | 2.77** |

|  | Weight Model 1 | | | Weight Model 2 | | | Weight+Fat Model 1 | | | Weight+Fat Model 2 | | |
| --- | --- | --- | --- | --- | --- | --- | --- | --- | --- | --- | --- | --- |
| **Factors** | **Estimate** | **±SE** | **t-value** | **Estimate** | **±SE** | **t-value** | **Estimate** | **±SE** | **t-value** | **Estimate** | **±SE** | **t-value** |
| (Intercept) | 2.31 | 0.27 | 8.66*** | 2.30 | 0.28 | 8.08*** | 2.29 | 0.27 | 8.57*** | 2.29 | 0.28 | 8.05*** |
| Age | -0.01 | 0.01 | -1.18 | -0.01 | 0.01 | -0.48 | -0.01 | 0.01 | -0.98 | 0.00 | 0.01 | -0.40 |
| Age^2^ | 0.00 | 0.00 | 0.70 | 0.00 | 0.00 | 0.48 | 0.00 | 0.00 | 0.52 | 0.00 | 0.00 | 0.39 |
| Sex | 0.43 | 0.06 | 6.77*** | 0.47 | 0.16 | 2.98** | 0.33 | 0.09 | 3.58*** | 0.41 | 0.17 | 2.36* |
| Forest | 0.06 | 0.07 | 0.85 | -0.52 | 0.10 | -5.06*** | 0.06 | 0.07 | 0.83 | -0.52 | 0.10 | -5.03*** |
| Riverine | -0.10 | 0.07 | -1.56 | -0.39 | 0.09 | -4.18*** | -0.10 | 0.07 | -1.52 | -0.39 | 0.09 | -4.19*** |
| Dry Season | 0.13 | 0.02 | 6.89*** | 0.04 | 0.06 | 0.62 | 0.13 | 0.02 | 6.89*** | 0.04 | 0.06 | 0.60 |
| Wet Season | -0.24 | 0.02 | -10.37*** | -0.19 | 0.11 | -1.78 | -0.24 | 0.02 | -10.37*** | -0.19 | 0.11 | -1.77° |
| Education | -0.01 | 0.01 | -0.86 | -0.01 | 0.01 | -0.66 | -0.01 | 0.01 | -0.89 | -0.01 | 0.01 | -0.68 |
| Spanish | 0.10 | 0.07 | 1.53 | 0.06 | 0.07 | 0.92 | 0.09 | 0.07 | 1.36 | 0.05 | 0.07 | 0.81 |
| **% Body Fat** |  |  |  |  |  |  | **-0.01** | **0.01** | **-1.42** | **-0.01** | **0.01** | **-0.85** |
| **Weight** | **0.00** | **0.00** | **0.60** | **0.00** | **0.00** | **0.83** | **0.01** | **0.00** | **1.33** | **0.01** | **0.00** | **1.17** |
| Age*Sex |  |  |  | -0.01 | 0.00 | -1.99* |  |  |  | -0.01 | 0.00 | -1.95° |
| Age*Dry Season |  |  |  | -0.01 | 0.00 | -4.56*** |  |  |  | -0.01 | 0.00 | -4.55*** |
| Age*Wet Season |  |  |  | 0.01 | 0.00 | 3.25** |  |  |  | 0.01 | 0.00 | 3.25** |
| Sex*Forest |  |  |  | 0.53 | 0.12 | 4.23*** |  |  |  | 0.52 | 0.12 | 4.17*** |
| Sex*Riverine |  |  |  | 0.26 | 0.12 | 2.12* |  |  |  | 0.26 | 0.12 | 2.15* |
| Sex*Dry Season |  |  |  | 0.26 | 0.04 | 6.92*** |  |  |  | 0.26 | 0.04 | 6.93*** |
| Sex*Wet Season |  |  |  | -0.35 | 0.05 | -7.46*** |  |  |  | -0.35 | 0.05 | -7.46*** |
| Forest*Dry Season |  |  |  | 0.52 | 0.05 | 11.25*** |  |  |  | 0.52 | 0.05 | 11.25*** |
| Riverine*Dry Season |  |  |  | 0.00 | 0.05 | 0.01 |  |  |  | 0.00 | 0.05 | 0.02 |
| Forest*Wet Season |  |  |  | -0.03 | 0.09 | -0.36 |  |  |  | -0.03 | 0.09 | -0.36 |
| Riverine*Wet Season |  |  |  | 0.25 | 0.09 | 2.78** |  |  |  | 0.25 | 0.09 | 2.77** |

° *p*<0.1, * *p*<0.05, ** *p*<0.01, *** *p*<0.001
