# Supplementary material for: Physical Activity and Modernization among Bolivian Amerindians
Source: PLoS One. 2013 Jan 31;8(1):e55679. doi: 10.1371/journal.pone.0055679 (PMC3561330; doi:10.1371/journal.pone.0055679)
Supplement: Table S3 — Same as Table 3 but sample excludes observations of logging activities. (DOCX) [file pone.0055679.s005.docx]

**SUPPLEMENTARY TABLE S3.** Same as Table 3 but sample excludes observations of logging activities

|  | Model 1 (AIC=1492.8) | | | Model 2 (AIC=1481.3) | | |
| --- | --- | --- | --- | --- | --- | --- |
| **Factors** | **Estimate** | **±SE** | **t-value** | **Estimate** | **±SE** | **t-value** |
| (Intercept) | 2.57 | 0.17 | 14.73*** | 2.64 | 0.17 | 15.14*** |
| Age | 0.00 | 0.00 | 0.51 | 0.00 | 0.00 | 0.61 |
| Age^2^ | 0.00 | 0.01 | -0.67 | 0.00 | 0.01 | -0.80 |
| Sex (male) | 0.20 | 0.03 | 5.83*** | 0.21 | 0.03 | 6.02*** |
| Forest (vs. Near Town) | -0.29 | 0.04 | -7.06*** | -0.42 | 0.05 | -8.68*** |
| Riverine (vs. Near Town) | -0.22 | 0.04 | -5.86*** | -0.31 | 0.04 | -7.52*** |
| Dry Season (vs. intermediate) | -0.03 | 0.01 | -2.24* | -0.12 | 0.02 | -6.01*** |
| Wet Season (vs. intermediate) | -0.03 | 0.02 | -1.49 | -0.11 | 0.06 | -1.74° |
| Education (highest grade) | 0.00 | 0.01 | 0.00 | 0.00 | 0.01 | -0.13 |
| Spanish (0-2) | 0.03 | 0.04 | 0.79 | 0.02 | 0.04 | 0.65 |
| BMI | -0.01 | 0.01 | -1.62 | -0.01 | 0.01 | -1.60 |
| Forest*Dry Season |  |  |  | 0.23 | 0.04 | 6.31*** |
| Riverine*Dry Season |  |  |  | 0.10 | 0.04 | 2.66** |
| Forest*Wet Season |  |  |  | 0.12 | 0.07 | 1.69° |
| Riverine*Wet Season |  |  |  | 0.21 | 0.07 | 2.88** |

° *p*<0.1, * *p*<0.05, ** *p*<0.01, *** *p*<0.001
