# Supplementary material for: Physical Activity and Modernization among Bolivian Amerindians
Source: PLoS One. 2013 Jan 31;8(1):e55679. doi: 10.1371/journal.pone.0055679 (PMC3561330; doi:10.1371/journal.pone.0055679)
Supplement: Table S4 — Regression models of daytime (a) physical activity ratios (PAR) and (b) BMI among women age 20–45. Linear mixed-model (LMM) of PAR uses behavioral observations as unit of analysis and includes random effects for individual and time sampled (cf. Tables 3). Ordinary least squares (OLS) regression of BMI uses individual means as unit of analysis (cf. Table 4), with reproductive state reflecting pregnancy or lactation status during time of anthropometric measurement. Controls include age, region and Spanish fluency. Baseline for region is “Near Town” and for reproductive state is “non-pregnant, non-lactating”. (DOCX) [file pone.0055679.s006.docx]

**SUPPLEMENTARY TABLE S4.** Regression models of daytime (a) physical activity ratios (PAR) and (b) BMI among women age 20-45. Linear mixed-model (LMM) of PAR uses behavioral observations as unit of analysis and includes random effects for individual and time sampled (cf. Tables 3). Ordinary least squares (OLS) regression of BMI uses individual means as unit of analysis (cf. Table 4), with reproductive state reflecting pregnancy or lactation status during time of anthropometric measurement. Controls include age, region and Spanish fluency. Baseline for region is “Near Town” and for reproductive state is “non-pregnant, non-lactating”.

|  | Model 1 PAR: | | | Model 2 BMI: | | |
| --- | --- | --- | --- | --- | --- | --- |
| **Factors** | **Estimate** | **±SE** | **t-value** | **Estimate** | **±SE** | **t-value** |
| Intercept | 2.32 | 0.11 | 21.62*** | 21.33 | 1.15 | 18.54 |
| Pregnant | 0.36 | 0.16 | 2.23* | 5.32 | 2.70 | 1.973* |
| Lactating | 0.10 | 0.14 | 0.70 | 2.79 | 2.86 | 0.975 |
| Pregnant*Age | -0.02 | 0.01 | -2.93** | -0.15 | 0.08 | -1.794° |
| Lactating*Age | 0.00 | 0.00 | -0.98 | -0.08 | 0.10 | -0.817 |
| Age | 0.00 | 0.00 | -0.27 | 0.07 | 0.03 | 1.998* |
| Region = Forest | -0.30 | 0.06 | -5.35*** | -1.03 | 0.60 | -1.715° |
| Region = Upriver | -0.26 | 0.05 | -5.37*** | -1.24 | 0.47 | -2.62** |
| Spanish Fluency | -0.02 | 0.04 | -0.55 | 0.56 | 0.33 | 1.695° |

° *p*<0.1, * *p*<0.05, ** *p*<0.01, *** *p*<0.001
