# Supplementary material for: Physical Activity and Modernization among Bolivian Amerindians
Source: PLoS One. 2013 Jan 31;8(1):e55679. doi: 10.1371/journal.pone.0055679 (PMC3561330; doi:10.1371/journal.pone.0055679)
Supplement: Table S5 — Predictors of body size. Same as Table 4, Model 2, but replaces BMI with other body size variables. (DOCX) [file pone.0055679.s007.docx]

**SUPPLEMENTARY TABLE S5.** Predictors of body size. Same as Table 4, Model 2, but replaces BMI with other body size variables

|  | **Fat-free mass** | | | **% Body Fat** | | | **Weight** | | | | | |
| --- | --- | --- | --- | --- | --- | --- | --- | --- | --- | --- | --- | --- |
| **Variable** | **Estimate** | **±SE** | **t-value** | **Estimate** | **±SE** | **t-value** | **Estimate** | **±SE** | | **t-value** | | |
| (Intercept) | 37.07 | 3.24 | 11.42*** | 11.01 | 5.25 | 2.10* | 43.60 | | 4.40 | | 9.91*** |  |
| PAR | 0.46 | 0.70 | 0.65 | -0.10 | 1.02 | -0.10 | 0.60 | | 0.89 | | 0.67 |  |
| Age (yrs) | 0.15 | 0.13 | 1.18 | 0.50 | 0.20 | 2.47* | 0.42 | | 0.17 | | 2.44* |  |
| Age^2^ | 0.00 | 0.00 | -1.65 | 0.00 | 0.00 | -2.05* | -0.01 | | 0.00 | | -3.07** |  |
| Sex (male) | 10.17 | 1.10 | 9.20*** | -10.99 | 3.74 | -2.94** | 4.30 | | 3.24 | | 1.33 |  |
| Forest | -1.11 | 0.89 | -1.25 | 9.96 | 3.70 | 2.69** | -0.96 | | 1.13 | | -0.85 |  |
| Riverine | -1.86 | 0.80 | -2.32* | 6.06 | 3.21 | 1.89° | -2.36 | | 1.02 | | -2.32* |  |
| Education (highest grade) | 0.36 | 0.16 | 2.32* | -0.21 | 0.23 | -0.93 | 0.19 | | 0.20 | | 0.94 |  |
| Spanish (0-2) | -1.78 | 1.01 | -1.77° | 8.24 | 1.49 | 5.54*** | 4.70 | | 1.30 | | 3.63*** |  |
| Age * Sex (male) |  |  |  | 0.10 | 0.08 | 1.28 | 0.09 | | 0.07 | | 1.32 |  |
| Age * Region (Forest) |  |  |  | -0.25 | 0.09 | -2.86** |  | |  | |  |  |
| Age * Region (Riverine) |  |  |  | -0.18 | 0.08 | -2.15* |  | |  | |  |  |
| Sex (male) * Spanish | 2.54 | 1.18 | 2.15* | -6.49 | 1.85 | -3.51*** | -2.44 | | 1.62 | | -1.51 |  |

° *p*<0.1, * *p*<0.05, ** *p*<0.01, *** *p*<0.001
